# Graph neural network based coarse-grained mapping prediction†

**DOI:** 10.1039/d0sc02458a

**Published:** 2020-08-11

**Authors:** Zhiheng Li, Geemi P. Wellawatte, Maghesree Chakraborty, Heta A. Gandhi, Chenliang Xu, Andrew D. White

**Affiliations:** Department of Computer Science, University of Rochester USA; Department of Chemistry, University of Rochester USA; Department of Chemical Engineering, University of Rochester USA andrew.white@rochester.edu

## Abstract

The selection of coarse-grained (CG) mapping operators is a critical step for CG molecular dynamics (MD) simulation. It is still an open question about what is optimal for this choice and there is a need for theory. The current state-of-the art method is mapping operators manually selected by experts. In this work, we demonstrate an automated approach by viewing this problem as supervised learning where we seek to reproduce the mapping operators produced by experts. We present a graph neural network based CG mapping predictor called Deep Supervised Graph Partitioning Model (DSGPM) that treats mapping operators as a graph segmentation problem. DSGPM is trained on a novel dataset, Human-annotated Mappings (HAM), consisting of 1180 molecules with expert annotated mapping operators. HAM can be used to facilitate further research in this area. Our model uses a novel metric learning objective to produce high-quality atomic features that are used in spectral clustering. The results show that the DSGPM outperforms state-of-the-art methods in the field of graph segmentation. Finally, we find that predicted CG mapping operators indeed result in good CG MD models when used in simulation.

## Abstract 2

We propose a scalable graph neural network-based method for automating coarse-grained mapping prediction for molecules.

## Graphical abstract

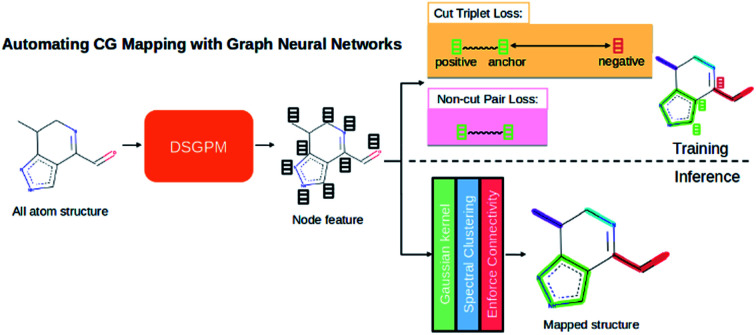

## 1 Introduction

Coarse grained (CG) models can be viewed as a two part problem of selecting a suitable CG mapping and a CG force field. In this work we focus on addressing the issue of CG mapping selection for a given system. A CG mapping is a representation of how atoms in a molecule are grouped to create CG beads. Once the CG mapping is selected, CG force field parameters required for the CG simulation can be determined *via* existing bottom-up^1^ or top-down^2^ CG methods. The former use atomistic simulations for parameterization of the CG force fields while the latter use experimental data.

Conventionally, a CG mapping for a molecule is selected using chemical and physical intuition. For example, the widely used MARTINI CG model uses mapping of four heavy (non-hydrogen) atoms to one CG bead as chosen by experts.^3^ Another popular choice of CG mapping for proteins and peptides is to assign one CG bead centered at the α-carbon for each amino acid. These choices are not built on any thermodynamic or theoretical argument. A recent discussion on commonly used mapping strategies is summarized in Ingólfsson *et al.*^4^ There have been recent efforts in developing systematic and automated methods in selecting a CG mapping for a molecule. Automation of CG mapping is important to enhance scalability and transferability.

Webb *et al.*^5^ proposed a spectral and progressive clustering on molecular graphs to identify vertex groups for subsequent iterative bond contractions that lead to CG mappings with hierarchical resolution. Wang and Gómez-Bombarelli^6^ developed an auto-encoder based method that simultaneously learns the optimal CG mapping of a given resolution and the corresponding CG potentials. Giulini *et al.*^7^ proposed a mapping entropy based method to simplify the model representation of biomolecules. Their theoretical model focuses on preserving most information content in the lower resolution model compared to the all atom model. Chakraborty *et al.*^8^ reported a hierarchical graph method where multiple mappings of a given molecule are encoded in a hierarchical graph, which can further be used to auto-select a particular mapping using algorithms like uniform-entropy flattening.^9^ In a recent systematic study on the effects of CG resolution on reproducing on and off target properties of a system, Khot *et al.*^10^ hypothesized that low-resolution CG models might be information limited, instead of having a representability limitation. This hypothesis suggests that there might be ways of enhancing the information of CG models without increasing their dimension and complexity. This is supported by a recent study of 26 CG mappings for 7 alkane molecules that found little correlation between mapping resolution and CG model performance.^11^ There is not only a lack of methods to compute mapping operators, there is no agreed upon goal in choosing mapping operators.

Mapping operators used in practice for CG simulations are usually rule-based,^3,4^ but recent advances have been made in algorithmic^5,8,12–15^ and unsupervised methods.^16^ Rule-based schemes have fixed resolution and must be created for each molecular functional group, limiting their application to sequence-defined biomolecules or polymers. Algorithmic and unsupervised methods have only been qualitatively evaluated on specific systems. The Chakraborty *et al.*,^8^ Gómez-Bombarelli *et al.*^16^ methods also required explicit molecular dynamics simulations, which leads to questions about hyperparameters (*e.g.*, sampling, atomistic force field) and requires at least hours per system. Such methods also are not learning nor optimizing mapping operator correctness directly. Supervised learning has not been used in previous work because there are no datasets and no obvious optimality criteria.

Here we have avoided the open question of “which is the best mapping?”, by choosing to match human intuition, the main selection method of past mapping operators. We demonstrate a supervised learning based approach using a graph neural network framework, Deep Supervised Graph Partitioning Model (DSGPM). To train and evaluate the DSGPM, we compiled a Human-annotated Mappings (HAM) dataset with expert annotated CG mappings of 1180 organic molecules, where each molecule has one or more coarse graining annotations by human experts. We expect this dataset can facilitate research on coarse graining and the graph partition problem. The HAM database allows DSGPM to learn CG mappings directly from annotations. Our framework is closely related to the problem of graph partitioning and has molecular feature extraction and embedding as major components. The graph neural network is trained *via* metric learning objectives to produce good atom embeddings of molecular graph, which creates better affinity matrix for spectral clustering.^17^ Should there be a consensus in the field on what are “best” mappings, our model can be easily adapted to match a new dataset of annotations.

## 2 Related work

### 2.1 Molecular feature extraction

The applications of graph convolutional neural networks (GCNN) to molecular modeling is an emerging approach for “featurizing” molecular structures. Featurizing a molecule is a challenging process which extracts useful information from a molecule to a fixed representation. This is important since conventional machine learning algorithms can accept only a fixed length input. However, a molecule can have arbitrary sizes and varying connectivities. GCNNs have become a useful tool for molecular featurization as they can be used for deep learning of raw representations of data which are less application specific unlike molecular fingerprints. Kearnes *et al.*^18^ have shown in their work that GCNNs can be used to extract molecular features with little preprocessing as possible. Furthermore, it is shown that the results from the GCNN are comparable to neural networks trained on molecular fingerprint representations. Wu *et al.*^19^ have implemented a GCNN featurization method in MoleculeNet. The GCNN is used to compute an initial feature vector which describe an atom's chemical environment and a neighbor list for each atom.^19^ Additionally, they show that unlike the fingerprints methods, GCNNs create a learnable process to extract molecular features using differentiable network layers. Gilmer *et al.*^20^ have developed a generalized message passing neural network (MPNN) to predict molecular properties. In this work, authors have used a GCNN to extract molecular features and to learn them from molecular graphs. The authors also state that there is a lack of a generalized framework which can work on molecular graphs for feature extraction. Given the proven success of GCNNs in feature extraction, the motivation for our work was to develop a generalized deep learning based method apt for chemistry problems.

### 2.2 Graph partitioning and graph neural network

If a molecule is viewed as a graph, the problem of selecting a CG mapping is analogous to partitioning the molecular graph. While there has been limited application of molecular graph for the purpose of selecting CG mappings, as discussed earlier, we would like to highlight some strategies employed for problems relevant to graph partitioning. Spectral clustering^17,21,22^ is one of the baseline method used in graph clustering task. Compared with Expectation–Maximization (EM),^23^ spectral clustering has a better modeling on pairwise affinity given by the adjacency matrix of a graph. METIS^24^ solves the graph partition problem in a multilevel scheme *via* coarsening, partition, and refinement steps. Graclus^25^ proposed a generalized kernel *k*-means method with better speed, memory efficiency, and graph clustering result. Fortunato^26^ has a comprehensive review of the methods developed for community detection in graphs. Safro *et al.*^27^ compared different graph coarsening schemes for graph partitioning using algebraic distance between nodes of the graph. Recently, some graph neural network^28^ based graph partitioning methods have been proposed. GAP^29^ uses graph neural networks to predict node-partition assignment probability, which is learned through normalized cut loss and balanced cut loss. ClusterNet^30^ adds differentiable *k*-means clustering at the end of graph neural network to enable end-to-end training. Compared to the aforementioned methods, our DSGPM combines the advantages of both spectral clustering and a graph neural network, leading to better results than either alone. We also propose and justify a novel metric learning loss to train the graph neural network.

### 2.3 Metric learning

The goal of the metric learning is to learn a model which encodes the input data to an embedding space, where embeddings (usually represented by fixed length vector) of similar data objects are separated by short distances in the embedding space and different data objects are separated by larger distances in the embedding space. Hadsell *et al.*^31^ proposed a siamese network trained *via* contrastive loss which (1) minimizes *L*_2_ distance for instances from the same group, and (2) maximizes *L*_2_ distance for instances from the different groups if the *L*_2_ distance is larger than a margin. Instead of only considering a pair of data, Schroff *et al.*^32^ considered a triplet of data 〈anchor, positive, negative〉 and triplet loss to ensure *L*(anchor, negative) (distance between anchor and negative) should be larger than *L*(anchor, positive) (distance between anchor and positive) by a margin. However, the methods above have only been applied to nonstructural data (*e.g.*, image clustering). Furthermore, one of challenging problem is sampling pairs or triplets of data from the dataset. In contrast, our proposed method can efficiently enumerate pairs or triplets by explicitly treating the graph structure.

## 3 Method

### 3.1 Problem formulation

Deep Supervised Graph Partitioning Model (DSGPM)§§The code for DSGPM can be accessed *via*https://github.com/rochesterxugroup/DSGPM. formulates the CG mapping prediction as a graph partitioning problem. Suppose *Q* is the set of atom types existing in the dataset. An atom in a molecule is represented as a one-hot encoding of its atom type. Similarly, a bond is represented as a one-hot encoding of its bond type (*e.g.*, single, double, aromatic, *etc.*). Therefore, a molecular with *n* atoms is formulated as a graph *G* = (*V*, *E*), where *V* ∈ ℝ^*n*×|*Q*|^ represents atoms and *E* ∈ ℝ^*n*×*n*×4^ denotes the adjacency matrix with encoded bond types.

### 3.2 Motivation

One strong baseline method to solve the graph partitioning problem is spectral clustering.^17^ The performance of spectral clustering is mainly decided by the quality of affinity matrix *S* ∈ ℝ^*n*×*n*^, where *S*_*ij*_ denotes the affinity (ranging from 0 to 1) between vertex *i* and vertex *j*. In this task, the adjacency matrix (ignoring bond type information in *E*) can serve as the affinity matrix fed into spectral clustering. However, for the CG mapping prediction problem, an ideal affinity matrix should have low affinity value of cut (edge connecting two atoms from different CG beads) and high affinity of an edge which is not a cut, while adjacency matrix only contains “0”s and “1”s to represent the existence of edges.

### 3.3 Deep supervised graph partitioning model

**Fig. 1:**
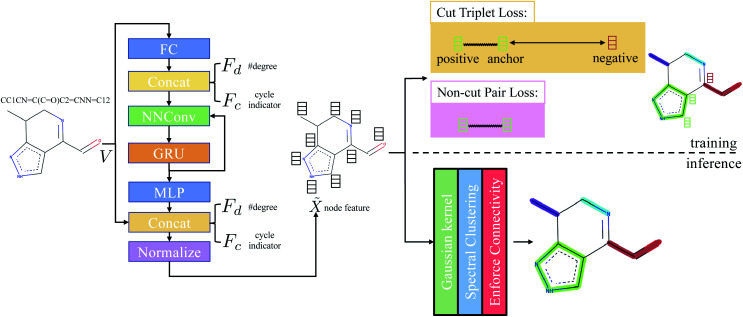
Overview of the method. Adjacency matrix *E* is omitted from the figure. FC stands for fully-connected layer and MLP stands for multilayer perceptron. Concat denotes concatenation. NNConv and GRU are explained in eqn (1) and (2), respectively. “Normalize” means *L*_2_ normalization.

The main difference from the baseline method is a graph neural network 
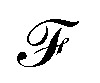
 that is used to obtain a better affinity matrix, where 
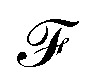
 follows the architecture design of MPNN.^20^ The overview of the method is shown in Fig. 1. With the molecular graph *G* as the input, 
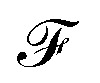
 extracts *q*-dimensional atom features *X̃* ∈ ℝ^*n*×*q*^ through message passing 
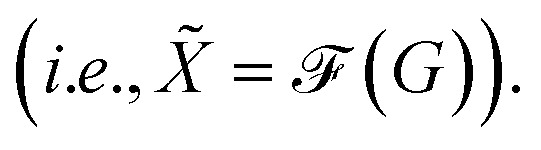
 Concretely, 
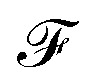
 first uses a fully-connected layer to project one-hot atom type encoding into the feature space. Then, we concatenate the embedded feature with two numbers: (1) number of degree; (2) cycle indicator (*i.e.*, whether the atom is in a cycle) (zero or one) to obtain *d*-dimensional feature *X*^0^ ∈ ℝ^*n*×*d*^. We find out that adding these two features improves the result (Sec. 4.5). Next, *X*^0^ is iteratively updated *T* time steps to obtain *X*^*T*^:1
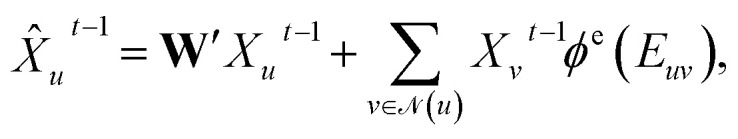

2 *X*_*u*_^*t*^ = GRU(*X̂*_*u*_^*t*−1^,*H*_*u*_^*t*−1^),

where underscript *u* denotes *u*-th atom and superscript *t* denotes time step; 
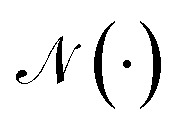
 denotes the set of neighboring nodes of the given vertex; **W** ∈ ℝ^*d*×*d*^ is a weight matrix; superscript ′ denotes transpose; *ϕ*^e^(·):{0,1}^4^ ↦ ℝ^*d*×*d*^ is function mapping bond type to edge-conditioned weight matrix, which is implemented as multilayer perceptron; GRU stands for gated recurrent unit.^33^ Finally, the output feature *X̃* is obtained by:

3 *X̃*′ = Concat(MLP(*X*^*T*^),*V*,*F*_d_,*F*_c_),

4
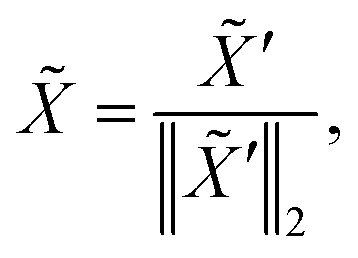
where Concat denotes concatenation; MLP denotes multilayer perceptron; *F*_d_ ∈ ℕ^*n*^ denotes degree of each atom and *F*_c_ ∈ {0,1}^*n*^ is cycle indicator (*i.e.* whether an atom is in a cycle).

After computing the atom feature *X̃*, the affinity matrix *A* ∈ ℝ^*n*×*n*^ can be calculated by a Gaussian kernel:5
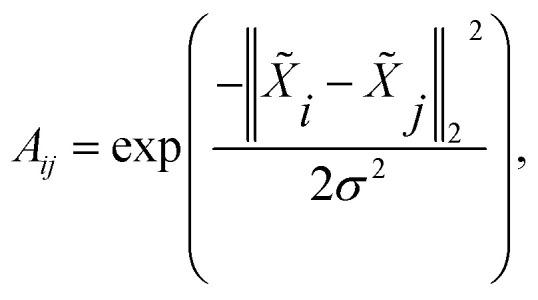
where *σ* is the bandwidth and is set to *σ* = 1 in the experiment.

Therefore, in order to obtain a good affinity matrix, ‖*X̃*_*i*_ − *X̃*_*j*_‖_2_ should be large when edge 〈*i*, *j*〉 is a cut and small when edge 〈*i*, *j*〉 is not a cut. Hence, by utilizing the ground-truth partition *B* ∈ ℕ^*n*^ (*B*_*i*_ denotes coarse grain (partition) index of *i*-th atom), we design cut triplet loss and non-cut pair loss to guide the network outputting good node feature *X̃* during training.

### 3.4 Training

#### 3.4.1 Cut triplet loss

The goal of cut triplet loss is to push pairs of node embeddings far away from each other when they belong to different partitions. To this end, we first extract all triplets from the given molecular graph *G* where each triplet contains three atoms: (anchor atom, positive atom, negative atom) denoted by {a,p,n} such that *B*_a_ = *B*_p_ but *B*_a_ ≠ *B*_n_ (see “green” features and “red” feature on top-right of Fig. 1). In other words, we extract non-cut edge 〈a, p〉 and cut edge 〈a, n〉 sharing one common vertex *a*. The set of triplets is denoted by *P*. Then, cut triplet loss is defined by:6
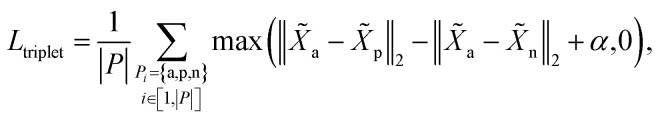
where *α* is a hyperparameter denoting the margin in triplet loss. By optimizing cut triplet loss, the objective ‖*X̃*_a_ − *X̃*_p_‖_2_ + *α* ≤ ‖*X̃*_a_ − *X̃*_n_‖_2_ can be satisfied for all triplets.

#### 3.4.2 Non-cut pair loss

The purpose of non-cut pair loss is to pull pairs of node embeddings as close as possible when they are from the same partition. Therefore, all pairs of node a and a′ are extracted when edge 〈a, a′〉 is not a cut. The set of pairs of node is denoted as *S*. Then, non-cut pair loss is defined by:7
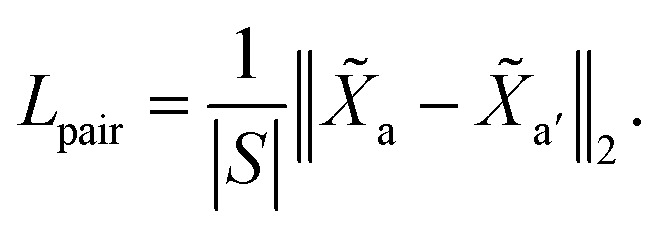


The final loss function to train the network is defined by:

8 *L* = *L*_triplet_ + *λL*_pair_,

where the coefficient is taken to be *λ* = 0.1.

### 3.5 Inference

In the inference stage, we first apply eqn (5) on the extracted node feature *X̃*. Then, based on affinity matrix *A*, spectral clustering is used to obtain the graph clustering result. Note that graph clustering is slightly different to graph partitioning. The latter requires the predicted partition must be a connected-component. Hence, we post-process the graph clustering result by enforcing connectivity of each graph partition: for each predicted graph cluster, if it contains more than one connected-component, we assign new indices to each connected-components.

## 4 Experiment

### 4.1 Dataset

Human-annotated Mappings (HAM) dataset¶¶HAM dataset can be downloaded *via*https://github.com/rochesterxugroup/HAM_dataset/releases. contains CG mappings of 1180 organic molecules with less than 25 heavy atoms. Each molecule was downloaded from the PubChem database as SMILES.^34^ One molecule was assigned to two annotators to compare the human agreement between CG mappings. These molecules were hand-mapped using a web-app. The completed annotations were reviewed by a third person, to identify and remove unreasonable mappings which did not agree with the given guidelines. Hence, there are 1.68 annotations per molecule in the current database (16% removed). To preserve the chemical and physical information of the all atom structure accurately, the annotators were instructed to group chemically similar atoms together into CG beads while preserving the connectivity of the molecular structure. They were also instructed to preserve the planar configuration of rings if possible by grouping rings into 3 or more beads.

### 4.2 Evaluation metrics

Adjusted Mutual Information (AMI)^35^ is used to evaluate the graph partition result in terms of nodes in the graph. Nodes from the same CG bead are assigned with the same cluster index and AMI compares predicted nodes' cluster indices with ground-truth nodes' cluster indices. We also evaluate graph partition result in terms of accuracy of predicting cuts from a graph. We report the precision, recall, and F1-score on cuts prediction (denoted by Cut Prec., Cut Recall, and Cut F1-score, respectively). Our method is trained and evaluated through 5-fold cross-validation^36^ to mitigate the bias of data split. Concretely, the dataset is split into 5 non-overlapping partitions (*i.e.*, one molecule only exists in one data partition). The experiment will run 5 iterations. At *i*-th iteration (*i* ∈ [1, 5]), the *i*-th split of the dataset is regarded as testing set (ground-truth partition *B* is not used) and rest 4 splits of the dataset is regarded as the training set (ground-truth partition *B* is used for training). Therefore, after training, DSGPM is evaluated on unseen molecules in the testing set. The final results are the average values over all iterations. Since one molecule may have multiple annotations, we choose one of the annotations that produces the best result for both AMI and cut accuracy.

### 4.3 Implementation details

DSGPM is trained with at most 500 epochs and we choose the epoch at which model achieves the best performance over the 5-fold cross validation.||||This setting is also used for comparison methods. The hidden feature dimension is 128. The implementation of spectral clustering used in the inference stage is from Scikit-learn.^37^ Since spectral clustering requires a hyperparameter to indicate the expected number of clusterings, we provide the ground-truth number of clusters based on CG annotations. Cycles of each molecular graph are obtained *via* “cycle_basis”^38^ function implemented by NetworkX.^39^ The code of graph neural network is based on PyTorch^40^ and PyTorch Geometric.^41^

### 4.4 Comparison with state-of-the-art

We compare our method with five state-of-the-art graph partitioning methods. We used officially released code of the comparing methods on HAM dataset. Here, we also show an alternative of our method (denoted by Cut Cls.): by regarding the graph partitioning problem of edge cut binary classification problem (*i.e.*, predicting the probability that an edge is a cut or not), we train DSGPM with binary cross-entropy loss. In the inference stage, we rank “cut probability” of each edge in descending order and take top-k edges as the final cut prediction, where *k* is the ground-truth number of cuts computed from the CG annotation. The result of comparison is shown in Table 1. The result shows that our method outperforms all state-of-the-art methods in terms of both AMI and cut accuracy. Moreover, DSGPM also outperforms Cut Cls., proving the effectiveness the metric learning training objectives and the importance of spectral clustering stage in our method. Additionally, by treating one annotation as prediction and the other annotation as ground-truth, we can show the agreement between different annotations (see last row in Table 1), which can be regarded as human annotator's performance. The result shows that our proposed DSGPM is very closed to human-level performance.

**Table 1:** Comparison with state-of-the-art methods. Average results over 5-fold cross validation are shown. Here, “Spec. Cluster.” means spectral clustering. The standard deviation of 5-fold cross-validation result under all evaluation metrics of our method is smaller than 0.01. Evaluation on human agreement (last row) is based on 128 molecules with 129 pairs of mappings, where mappings in each have the same number of CG beads

Method	AMI	Cut Prec.	Cut Recall	Cut F1-score
GAP^29^	0.33	0.47	0.73	0.54
Graclus^25^	0.45	0.58	0.81	0.65
ClusterNet^30^	0.52	0.64	0.62	0.58
METIS^24^	0.56	0.63	0.56	0.58
Cut Cls.	0.67	0.75	0.73	0.73
Spec. Cluster.^17^	0.73	0.75	0.75	0.75
**DSGPM** (ours)	**0.79**	**0.80**	**0.80**	**0.80**
Human	0.81	0.81	0.81	0.81

### 4.5 Ablation study

We study the contribution of degree and cycle indicator in the input. The results are shown in Table 2. Degree feature (w/o *F*_c_ in Table 2) improves the edge-based metrics (cut precision, cut recall, cut F1-score) and cycle indicator (w/o *F*_d_ in Table 2) contributes to all evaluation metrics. Combining both input feature boosts the performance further.

**Table 2:** Ablation study on the input of DSGPM. *F*_d_ and *F*_c_ denote number of degree and cycle indicator, respectively

Input	AMI	Cut Prec.	Cut Recall	Cut F1-score
w/o *F*_d_ & *F*_c_	0.781	0.797	0.801	0.798
w/o *F*_c_	0.783	0.800	0.803	0.801
w/o *F*_d_	0.790	0.806	0.807	0.806
**DSGPM**	**0.790**	**0.806**	**0.809**	**0.807**

We also examined the contribution of each loss terms, cut triplet loss and non-cut pair loss. The result in Table 3 shows that cut triplet loss plays the major role in the training objective and combining both loss terms will produce better performance, which proves that *L*_triplet_ and *L*_pair_'s objectives, separating atoms connected by an cut edge and concentrating features of atoms from the same partition, are reciprocal during training.

**Table 3:** Ablation study on loss terms. *L*_triplet_ denotes cut triplet loss and *L*_pair_ denotes non-cut pair loss

Loss terms	AMI	Cut Prec.	Cut Recall	Cut F1-score
w/o *L*_triplet_	0.73	0.75	0.76	0.7
w/o *L*_pair_	0.78	0.80	0.80	0.80
**DSGPM**	**0.79**	**0.80**	**0.80**	**0.80**

Furthermore, we study the impact of different values for the hyperparameters *λ* (see eqn (8)) and *σ* (see eqn (5)) in Tables 4 and 5, respectively. The ablation results show that DSGPM is not sensitive to changes of *λ* and choosing *σ* = 1 yields best results.

**Table 4:** Ablation study on loss terms. *λ* denotes the coefficient for non-cut pair loss (eqn (8))

*λ*	AMI	Cut Prec.	Cut Recall	Cut F1-score
0.1	0.79	0.80	0.80	0.80
0.5	0.78	0.80	0.80	0.80
1	0.78	0.80	0.80	0.80
2	0.78	0.80	0.80	0.80
10	0.78	0.80	0.80	0.80

**Table 5:** Ablation study on bandwidth of Gaussian kernel. *σ* denotes the bandwidth for Gaussian kernel in eqn (5)

*σ*	AMI	Cut Prec.	Cut Recall	Cut F1-score
0.5	0.77	0.79	0.79	0.79
1	0.79	0.80	0.80	0.80
1.5	0.77	0.79	0.79	0.79
2	0.76	0.78	0.78	0.78

### 4.6 Visualization

#### 4.6.1 CG mapping result

**Fig. 2:**
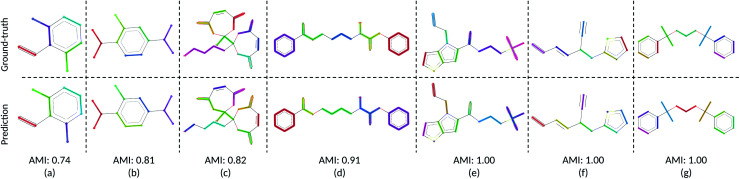
Visualization of the CG mapping prediction and the ground-truth. Atoms and corresponding edges that belong to the same CG bead are highlighted with the same color. Cut edges are not highlighted (*i.e.*, in black). Figures (a)–(g) illustrate the agreement between ground-truth data and the prediction. This similarity is measured using AMI. Note that colors between prediction and ground-truth may not match since colors are randomly selected.

We visualize the CG mapping prediction results against ground-truth in Fig. 2. Predicted mappings (e)–(g) are indistinguishable from the human annotations. Even though AMI values of structures (a)–(c) are comparatively lower, our predictions in (a)–(c) are still able to capture the essential features such as functional groups and ring conformations from the ground truth mappings. (a), (b), (e)–(g) also show that when rings in molecules are grouped into three CG beads by the human annotators, DSGPM model is able to capture this pattern. When rings are grouped into one CG bead (Fig. 2(d)), the model similarly chose this. Overall this shows that DSGPM can reproduce mappings which are significantly close to the human annotations. We have further compared our predictions with the widely used MARTINI mapping scheme. Results are shown in Fig. S3 in the ESI.†

#### 4.6.2 SARS-CoV-2 structure prediction

**Fig. 3:**
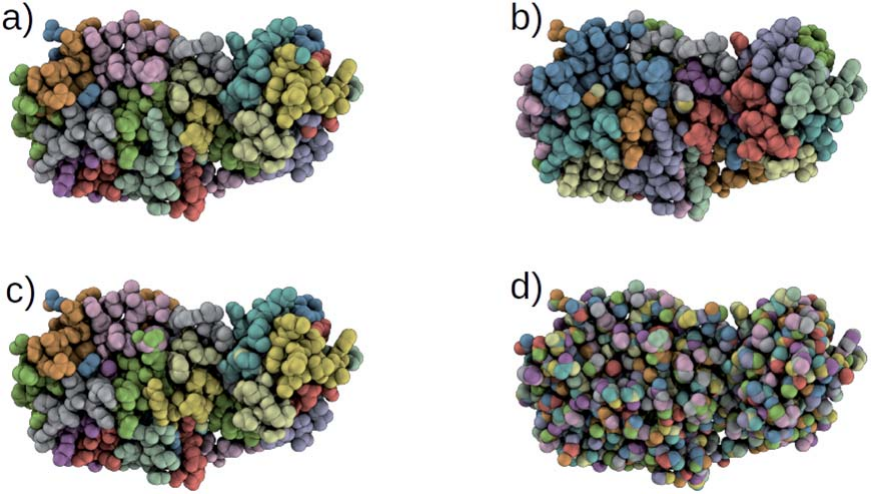
Comparison of CG mappings of SARS-CoV-2 protease structure predicted by baseline methods, (a) our DSGPM model (b) METIS^24^ (c) spectral clustering^17^ (d) Graclus.^25^ (a–c) have 32 CG beads while (d) contains 1455 CG beads.

Using our trained DSGPM, we predict the CG mappings for previously unseen SARS-CoV-2 protease structure (PDB ID: 6M03 (ref. 42)). In Fig. 3 we compare our result with three baseline methods. Even though our training dataset did not contain peptide sequences we show that our model is capable of predicting CG mappings of complex proteins. We see in Fig. 3 that our prediction is similar to predicted mapping from the spectral clustering method. This is an expected result as we use spectral clustering in the inference stage of our model. In spectral clustering, METIS methods and our model the resolution of the CG mapping can be controlled as the number of partitions is a hyper parameter. Mappings predicted by these three methods in Fig. 3 contain 32 beads. However, in the Graclus method the resolution cannot be controlled. In Fig. 3d, the predicted mapping from Graclus method contain 1455 CG beads. This is not a reasonable prediction as the fine grain structure contains 2367 atoms.

To gain a better understanding of the mappings, in Fig. S1 in the ESI,† we use the FASTA representation of the SARS-CoV-2 protease and color each one-letter code by the color of the CG beach to which each alpha-carbon belong. We see that our model is able to group amino acids with reasonable cuts along the backbone of the protein. Our model and spectral clustering method group 7–11 amino acids while the METIS method group 2–11 amino acids into CG beads. This shows that while DSGPM is capable of predicting state-of-the-art mapping for small molecules it can also be scaled to predict reasonable mappings for arbitrarily large structures.

#### 4.6.3 Model performance in CG simulations

**Fig. 4:**
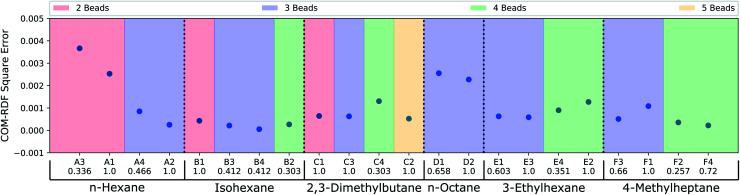
COM-RDF square errors as previously reported for CG mappings of 6 alkane molecules.^11^ The mappings for each molecule have been categorized into colored blocks corresponding to the number of CG beads. For each block, the mappings are arranged in the order of increasing AMI values, as indicated below the CG mapping labels.

Thus far, the model has been judged against human-annotated mappings and not in molecular dynamics simulation. To assess the predicted mappings, we draw upon the simulation results from recent work by Chakraborty *et al.*^11^ where force matching was used for coarse-graining. We have compared the performance of the CG mappings predicted by DSGPM for 6 alkane molecules with multiple CG bead numbers, giving 22 different simulation results. The individual mappings of the 6 alkane molecules (*n*-hexane, isohexane, 2,3-dimethylbutane, *n*-octane, 3-ethylhexane, and 4-methylheptane) that were considered in Chakraborty *et al.*^11^ and those predicted by DSGPM are shown in Fig. S2.† DSGPM predicts one mapping per molecule/bead number. To assess the quality of these mappings, we show how the CG simulation error changes for mappings other than the predicted DSGPM mapping as measured by AMI. Decreasing error as a AMI increases (better performance as we get closer to the DSGPM prediction) indicates good model performance. Fig. 4 shows the square errors for center-of-mass (COM) radial distribution function (RDF) relative to the all atom simulation as previously reported^11^ for each of the 6 alkane molecules. For a given molecule, the mappings are categorized into colored blocks corresponding to the number of beads in the CG mapping. AMI values of the mappings are computed relative to the CG mappings from DSGPM with the same number of CG beads. The mappings within the same colored block are arranged in increasing order of AMI values. It is observed that for most of the alkanes, a mapping with higher AMI compared to another with equal number of beads, yields lower COM-RDF square error (6 instances). 4 bead 3-ethylhexane mappings and 3 bead 4-methylheptane mappings are the only instances where a mapping with higher AMI gives higher COM-RDF square error than a comparable mapping with lower AMI. Thus the mappings predicted by DSGPM have good performance when used in simulations as judged from this small dataset of 22 simulations.

## 5 Conclusion

In this work, we propose a novel DSGPM as a supervised learning method for predicting CG mappings. By selecting good inputs and designing novel metric learning objectives on graph, the graph neural network can produce good atom features, resulting in better affinity matrix for spectral clustering. We also report the first large-scale CG dataset with experts' annotations. The result shows that our method outperforms state-of-the-art methods by a predicting mappings which are nearly indistinguishable from human annotations. The ablation study found that the novel loss term is the key innovation of the model. Furthermore, we show that our automated model can be used to predict CG mappings for macromolecules even though the training set was of small molecules and the CG mappings do result in good performance when implemented in force-matched CG simulations.

## Conflicts of interest

There are no conflicts to declare.

## Supplementary Material

SC-011-D0SC02458A-s001

SC-011-D0SC02458A-s002

SC-011-D0SC02458A-s003

SC-011-D0SC02458A-s004

SC-011-D0SC02458A-s005
