# Correction: Graph neural network based coarse-grained mapping prediction

**DOI:** 10.1039/d1sc90186a

**Published:** 2021-08-27

**Authors:** Zhiheng Li, Geemi P. Wellawatte, Maghesree Chakraborty, Heta A. Gandhi, Chenliang Xu, Andrew D. White

**Affiliations:** Department of Computer Science, University of Rochester USA; Department of Chemistry, University of Rochester USA; Department of Chemical Engineering, University of Rochester USA andrew.white@rochester.edu

## Abstract

Correction for ‘Graph neural network based coarse-grained mapping prediction’ by Zhiheng Li *et al.*, *Chem. Sci.*, 2020, **11**, 9524–9531, DOI: 10.1039/D0SC02458A.

The authors regret that eqn (5) was missing the adjacency matrix term. The correct form of eqn (5) is shown below:5
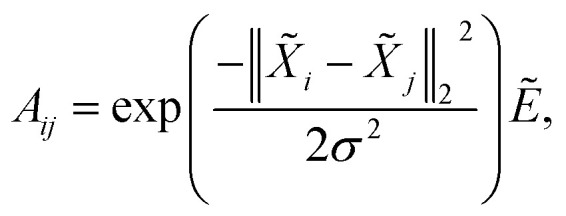
where *σ* is the bandwidth and is set to *σ* = 1 in the experiment. 
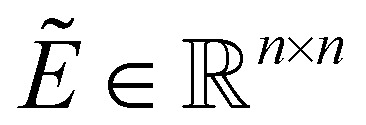
 denotes the adjacency matrix (*Ẽ*_*ij*_ = 1 if atom *i* and atom *j* are bonded, otherwise *Ẽ*_*ij*_ = 0).

The Royal Society of Chemistry apologises for these errors and any consequent inconvenience to authors and readers.

## Supplementary Material

