# Supplementary material for: Graph neural network based coarse-grained mapping prediction
Source: Chem Sci. 2020 Aug 11;11(35):9524–31. doi: 10.1039/d0sc02458a (PMC8161155; doi:10.1039/d0sc02458a)
Supplement: SC-011-D0SC02458A-s005 [file SC-011-D0SC02458A-s005.pdf]

Electronic Supplementary Material (ESI) for Chemical Science.
This journal is © The Royal Society of Chemistry 2020

Supplementary Information: Graph Neural Network based Coarse-Grain Mapping Prediction

Zhiheng Li,[a‡] Geemi P. Wellawatte,[b‡] Maghesree Chakraborty,[c] Heta A. Gandhi,[c] Chenliang Xu,[a*] and Andrew D. White,[c*]

[a] *Department of Computer Science, University of Rochester, USA*
[b] *Department of Chemistry, University of Rochester, USA*
[c] *Department of Chemical Engineering, University of Rochester, USA*
‡ *These authors contributed equally to this work*

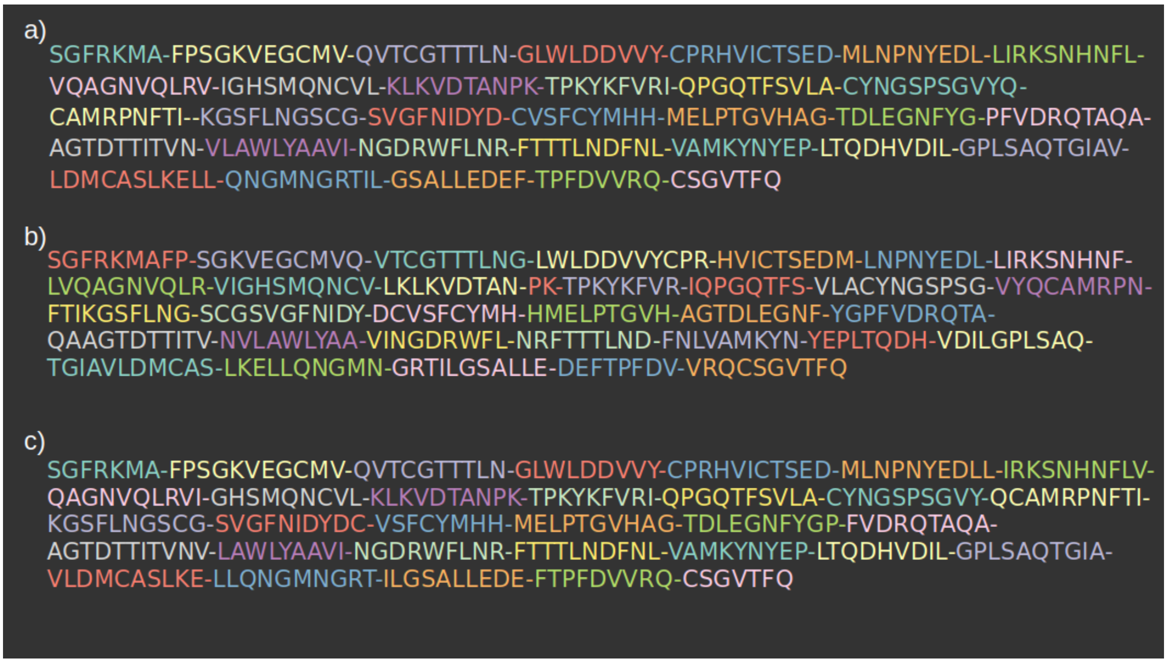

Fig. S1: Comparison of FASTA representations of the SARS-CoV-2 main protease coarse grained mappings predicted by DSGPM model with baseline methods. Predicted mappings from a) our DSGPM model b) METIS[1] and c) Spectral clustering[2] are illustrated. All three mappings presented here have 32 CG beads. We have colored each one-letter label of amino acids by the color of CG bead to which each alpha-carbon belong.

Molecule	Number of CG Beads	Predicted CG Mapping	Previously Reported CG Mappings
n-Hexane	2<br>3	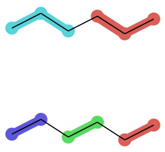	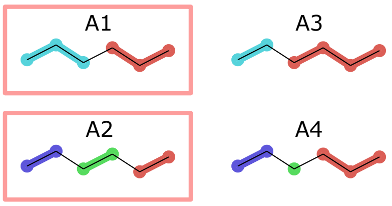
Isohexane	2<br>3<br>4	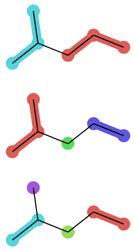	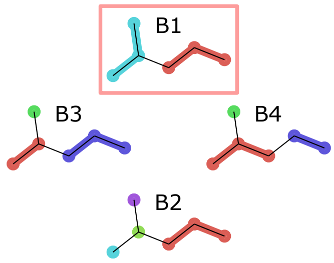
2,3-Dimethylbutane	2<br>3<br>4<br>5	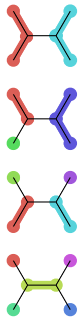	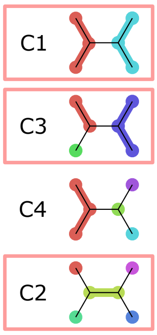
n-Octane	3	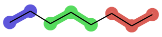	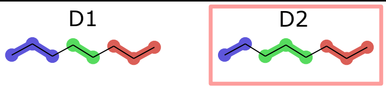
3-Ethylhexane	3<br>4	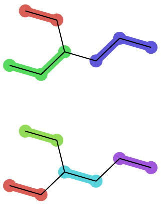	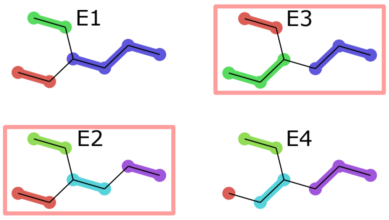
4-Methylheptane	3<br>4	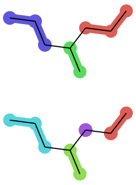	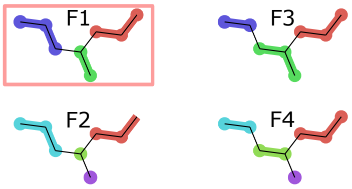

Fig. S2: Illustrations of previously studied CG mappings of 6 alkane molecules[3] (n-hexane, isohexane, 2,3-dimethylbutane, n-octane, 3-ethylhexane, and 4-methylheptane) and DSGPM predicted CG mappings of the 6 molecules with varying CG bead number. The mappings enclosed in red boxes correspond highlight instances where the predicted mappings are identical to one of previously studied mappings with the same number of CG beads and hence have AMI value as 1.

Peptide Sequence Mapping

We have considered 4 penta-peptides to compare the predicted CG mappings from DSGPM to the corresponding MARTINI CG models. The amino-acid sequence for the 4 peptides is of the form GGXGG, where G is glycine and X is either alanine (A), valine (V), aspartic acid (D) or tyrosine (Y). Note that peptides are previously unseen by the DSGPM model. For each of the peptides, we set the partition number hyperparameter for DSGPM to be equal to the number of CG beads in its MARTINI CG model. The MARTINI CG models for G and A have one bead each, those for V and D have two beads each and the CG model for Y has 4 beads. Hence, the number of partition hyperparameter for DSGPM was set as 5 for GGAGG, 6 for GGVGG and GGDGG, and 8 for GGYGG. Fig. S3 shows the predicted CG mappings along with the MARTINI CG models for the 4 penta-peptides. The predicted CG mappings closely mirror the MARTINI models for GGAGG, GGVGG and GGDGG, albeit with some deviations. The most prominent difference between the predicted result and the MARTINI CG model is observed for GGYGG.

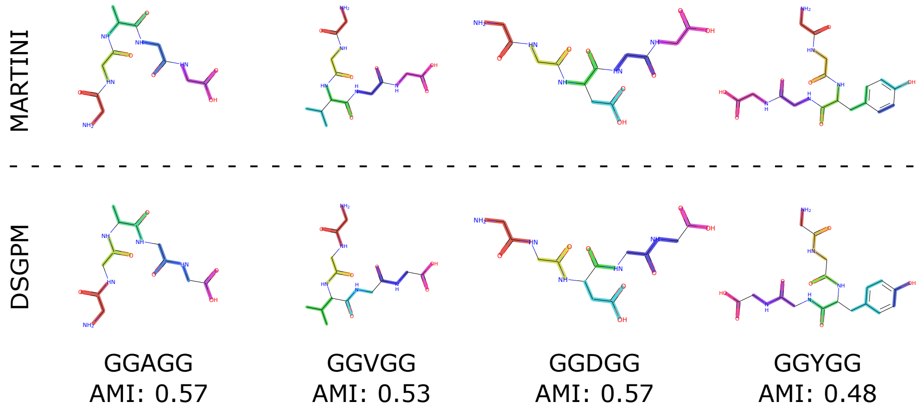

Fig. S3: Visualization of CG mappings for 4 peptides. We compare the CG mappings predicted by DSGPM to the corresponding mappings determined by the widely used MARTINI method. Atoms and their adjacent bonds belonging to the same CG bead are highlighted with the same color.

References

[1] G. Karypis and V. Kumar, *SIAM Journal on Scientific Computing*, 1998, **20**, 359–392.

[2] A. Y. Ng, M. I. Jordan and Y. Weiss, Advances in Neural Information Processing Systems, 2002, pp. 849–856.

[3] M. Chakraborty, J. Xu and A. D. White, *Phys. Chem. Chem. Phys.*, 2020, **22**, 14998–15005.
